# RE: Effects of Covid-19 on male reproductive system

**DOI:** 10.1590/S1677-5538.IBJU.2020.1113

**Published:** 2021-01-20

**Authors:** Rujittika Mungmunpuntipantip, Viroj Wiwanitkit

**Affiliations:** 1 Private Academic Consultant Bangkok Thailand Private Academic Consultant, Bangkok Thailand.; 2 Dr. DY Patil University Pune India Dr. DY Patil University, Pune, India.

*To the editor*,

We would like to share ideas on the report “Effects of Covid-19 on male reproductive system (1). Groner et al. concluded that “As demonstrated in other viral diseases, involvement of the male reproductive system is a possibility and it may reveal a new route of transmission and/or repercussions on its functions” (1). The impact of the new coronavirus on the reproductive system is an interesting issue. There might be 2 possible ways, direct or direct attacks of the reproductive system. Whether the SARS CoV2 virus can cross the cellular barrier to affect male reproductive cell is interesting. For female, it is approved that the virus cannot cross the barrier or pass the placenta to cause vertical transmission because the pathogen is too large to penetrate thorough cellular barrier (2). This might be the same phenomenon explaining no observation of the new virus existence in semen. There is also no evidence that the virus can be transmitted via sexual contact (3).

Regarding the possible indirect pathological process, the effect of high fever and alteration of endocrinological function has to be further studied. Nevertheless, since the virus infection usually has a short period of illness, there should not be much impact. It still requires a long- term follow-up on an infected patient. The evaluation of fertility in post-COVID-19 phase is interesting.

The Authors
